# Practical tips for moving your patient panel online

**DOI:** 10.12688/mep.19613.1

**Published:** 2023-03-24

**Authors:** Dimitrios Papanagnou, Nethra Ankam, Jordan Feingold-Link, Maria Poluch, Jared Kilpatrick, Andres Fernandez, Urvashi Vaid, Deborah Ziring

**Affiliations:** 1 Department of Emergency Medicine, Sidney Kimmel Medical College at Thomas Jefferson University, Philadelphia, PA, 19107, USA; 2 Department of Rehabilitation Medicine, Sidney Kimmel Medical College at Thomas Jefferson University, Philadelphia, PA, 19107, USA; 3 Departments of Emergency Medicine and Medical Education, Western Michigan University Homer Stryker, MD School of Medicine, Kalamazoo, MI, 49007, USA; 4 Department of Neurology, Sidney Kimmel College at Thomas Jefferson University, Philadelphia, PA, 19107, USA; 5 Department of Medicine, Sidney Kimmel Medical College at Thomas Jefferson University, Philadelphia, PA, 19107, USA

**Keywords:** undergraduate medical education, patient panels, virtual learning, technology, inclusive learning

## Abstract

Patient panels are an inspiring, highly rated educational tool to complement course goals and objectives for students in medical education programs. The COVID-19 pandemic brought challenges on the ability to successfully host in-person patient panels. These challenges resulted in the need to pivot in-person patient panels to online platforms, while still ensuring the quality and intimacy of patient narratives. In this 12 tips article, we share lessons learned in transitioning patient panels in our health systems science curriculum to an online experience for students enrolled in a pre-clinical medical education program.

## Introduction

Patient involvement in medical education is well described in the literature, with formal integration of patients into health professions education dating back to the 1960s ( Towle *et al*., 2010). There is a recent trend to increase exposure to patient experiences in the pre-clinical curriculum to develop empathy in students, provide a deeper understanding of patient illnesses, and model effective communication strategies with patients ( Gordon *et al*., 2020; Towle *et al*., 2010).

One approach to increase exposure to the patient experience is through the patient panel, where students listen to a small set of patients who are invited to share their respective experiences in navigating the health system and respond to questions posed by either faculty facilitators or members of the student audience. This approach has been consistently shown to be a positive emotional and learning experience for medical students and residents ( Colbert *et al*., 2009; Lenton & Storr, 2015; Salerno-Kennedy *et al*., 2009; Vail *et al*., 1996), with patients also reporting on the positive experience of being involved in training future clinicians ( Jackson *et al*., 2003). Effectively hosting patient panels, however, comes with its own set of challenges, including coordinating patient and student availability; securing patient transportation and hosting spaces; and assuring psychological safety for patients sharing their sensitive and deeply personal information.

The patient panel experience at our medical school was adapted to the virtual environment in 2020 due to the COVID-19 pandemic. Even with these changes, patient panels remained one of the most highly rated experiences students participated in during their first year of medical school. Through this process, we discovered unique benefits that were not previously considered. Coordinating availability became easier, transportation and venue space were no longer a concern, student engagement was amplified through the use of the chat function, and our invited patient panelists felt an increased sense of psychological safety, with the added ability to ‘remove themselves’ from the conversation at any given time.

Based on our experiences with this transition, we intend to continue to virtually host patient panels going forward, and we encourage other educators to consider this option. In this article, we share twelve tips for transitioning patient panels online to ease the planning and delivery process, and to mitigate potential challenges that may be encountered.

## Planning considerations

### Tip 1: Identify the goals of the patient panel

Well-designed patient panels that align with the goals of a curriculum have the potential to effectively contextualize coursework in undergraduate medical education within the larger healthcare system and enhance students’ understanding of specific topic areas ( Voelker, 2003). Therefore, it is essential that clear and concise learning objectives are first formulated for each session during the early stages of planning.

Learning goals should be appropriate for the students’ level of training and should be congruent with the timeline of how specific content areas are presented to the students in the curriculum ( Haras *et al*., 2021). For example, a patient panel designed to describe the experiences of patients living with diabetes mellitus could include goals ranging from initial diagnosis and management; interactions with the healthcare system; access to or lack of access to medications and supplies; their experiences with complications; their individual lifestyle changes and its effect on their families or their advocacy for other patients with diabetes. It is unrealistic, however, to cover this much content in a single panel, highlighting the importance of prioritizing the discussion around a few specific learning objectives that can be achieved during a single session.

Once the goals of the panel are defined, recruiting appropriate patients who can speak to those goals is a vital next step. For example, a patient recently diagnosed with mild type 2 diabetes mellitus without any specific disease-related complications may not be the ideal person for selection if the goal of the session is focused on managing the complications of diabetes mellitus.

Once the goals of the patient panel are identified and appropriate patients who can speak to these goals are recruited, it is essential to list the teaching points that will form the structure of the patient panel discussion. These points may be considered as prompts that generate patient responses geared to link to the goals of the panel. During this process, all questions should be intentionally designed and scripted. If time allows, the questions can be piloted in advance to ensure language clarity, so that the response elicited is appropriate for the panel discussion. Finally, less is more. A panel lasting forty-five minutes to an hour with three to four patient panelists is ideal and will ensure a rich discussion with an opportunity for all panelists to speak.

### Tip 2: Familiarize yourself with the technology

Decide early on the video platform you will use. There are many good options, but by committing early to one option, you allow everyone involved to become familiar with the technology. Many video services have online instructional videos, available free of charge, which provide instruction on many of the tools required for running a smooth panel. Choosing a tool familiar to your participants and audience might be an advantage, so consider video platforms that are commonly used at your institution for virtual sessions.

Encourage facilitators to have access to and practice key actions associated with the technology. Ensure facilitators running the panel are made hosts or co-hosts with full access to the platform settings and functions. These functions include ensuring that the platform accessibility options are in place (e.g., close captioning) and that they have the appropriate permissions to easily mute participants who are making distracting noises or remove any uninvited guests who may find their way onto the session. Facilitators should feel comfortable using the chat function to moderate questions and to communicate with the audience. They should also be able to ‘pin’ or ‘spotlight’ specific participants, which will allow the audience to view the same speaker.

The network bandwidth requirements of the chosen platform should also be considered. While smaller virtual meetings run smoothly with all participants on a single screen, a large group consumes a significant amount of network bandwidth, particularly when using video capabilities. Under such circumstances, the connection speed may be slow and cause interruptions in the service. In this case, all participants should be asked to disable their video if they are not in a speaking role.

### Tip 3: Prepare your facilitators for their assigned roles

When possible, several facilitators should be invited to assist with the delivery of the virtual patient panel. Each facilitator should have a specific, predetermined role. In our experience, we have found that at least two facilitators are ideal for a virtual panel.

The primary facilitator should be responsible for introductions and guiding the conversations, while a secondary facilitator should be responsible for managing the technological platform during the session, including chat moderation and troubleshooting. The latter role should not be taken lightly: someone should be readily available to mute participants when needed to mitigate distractions, and available to invited panelists who may require technological and last-minute assistance in the background.

The secondary facilitator assigned to moderate the chat function should identify their role to the student participants and give clear instructions on how the chat function will be used during the session. The facilitator should ensure that the chat stays within the boundaries of the primary discussion to avoid “side conversations” that create parallel discussion threads. The timing of answering the questions posed by the student participants should be determined in advance and coordinated with the primary facilitator (i.e., outline if questions will be answered as they come or if they will be answered at specific points during the session.).

## Patient panelist considerations

### Tip 4: Recruit a diverse panel of patients

Diversity in the patient panel participants is important to expose students to different patient narratives and perspectives. Diversity includes, but is not limited to age, sex, gender, disability, culture, race, and socioeconomic status. The online format has the potential to enhance diversity by allowing participation of patients outside of the local setting or patients with travel limitations. 

Barriers to patient diversity in the online setting must also be considered. These include access to technology, including equipment and reliable Internet access, which may unintentionally select for patients of higher socioeconomic status. In order to overcome the potential for reduction in socioeconomic diversity, schools should consider implementing mechanisms for decreasing technological barriers, including lending technology to patients (e.g., electronic tablets or WiFi hotspots) to allow for their participation.

### Tip 5: Review the technology with patients before the session

While Internet connection is ubiquitous, access to it and connectivity are not. Patients come from various backgrounds and may either lack the access to the technology, or the detailed knowledge required to navigate all the technological nuances of the virtual environment. Additionally, they may have physical and/or other limitations that preclude them from being able to use technology.

You should ensure that invited patient panelists are able to adequately access and use the technology required for their participation in the panel to avoid the frustrating consequences of technical failures (e.g., a microphone that will not unmute or a camera that will not turn on). Discussing the different tools that will be used can help avoid technical issues during the sessions; this includes reviewing the log-in process into the virtual panel platform (including reviewing the links), and ensure microphone and camera functionality.

Take the time to preempt connectivity and technical issues with a careful review of the technology with patients recruited for the panel at least several days before the session. This will allow for ample time to secure any assistance required for finding a solution. Additionally, a direct line of private communication (e.g., SMS text or private chat through the platform) should be established between the patients and the facilitator(s) prior to the session to address any needs that may arise throughout the process.

Finally, if possible, ensure that patients have access to a quiet and private location for the actual session and have time built into planning for a pre-brief before the session to appropriately set-up for the panel and time for a debrief after the session.

### Tip 6: Highlight functions of the technology that will support patients’ psychological safety

Although patients generally enjoy participating in patient panels to assist in the instruction of students and feel appreciated for their expertise for the respective conditions they live with, concerns have been raised regarding their possible exploitation ( Stacy & Spencer, 1999). The virtual platform, however, offers unique opportunities to protect their psychological safety and mitigate any feelings of exploitation.

Prior to the virtual panel, patients should be given the permission to mute themselves or turn off their camera at any time for any reason over the course of the session. The direct line of private communication described above will also afford patients the ability to express any concerns discretely to the facilitator(s) during the session. Facilitators may also consider establishing a verbal or non-verbal safety cue with patients to signal any discomfort during the conversation. This will allow the facilitator(s) to direct the discussion away from the patient panelists and give them the opportunity to take a break and/or excuse themselves from the session.

### Tip 7: Empower patients to share a holistic version of themselves

Patients are true experts of their condition(s) and should be empowered to share in the discussion a holistic version of themselves – a version that includes all opportunities and challenges of their daily lives. Patient perspective videos have already been shown to be an effective teaching tool for medical students ( Leeds *et al*., 2020). As expected, however, the virtual platform physically limits the visibility of the patients. Typically, only patients’ faces are visible on a virtual platform.

To provide a more holistic representation of themselves, if they are comfortable, patients can be encouraged to share photos or videos of themselves from their everyday environment. Patients can also be invited to share several of the social contexts of living with their respective condition(s). Patients living with physical disabilities, for example, can discuss some of their everyday challenges with navigating their day-to-day activities. It is important that panel moderators view patients as experts of their own lives and experiences, and should empower them to teach students about their own lives, rather than simply putting them on display. 

## During the session 

### Tip 8: Review online patient panel etiquette with students

Audience members can set the tone for the patient panel based on their virtual etiquette. It is important to review the expectations for proper patient panel etiquette. At the beginning of the panel, students should be prompted to turn on their cameras to welcome the invited patient panelist(s) and demonstrate their engagement. Students should be dressed professionally, with, potentially, a white coat when turning on their cameras. During the welcome and throughout the session, ensure that all audience participants are muted to prevent any unintended distractions.

Interactions between the patient(s) and students are critical to the panel. Before beginning the panel, review with students the types of questions that are permissible to ask patients, with the goal of avoiding insensitive questions. During the panel, direct students to post their questions in the chat. The moderator can then read the questions to the panelists to maintain the flow of the discussion. The moderator should provide students with the second option of messaging questions directly to the moderator. The latter option allows any uncomfortable and/or unsure students to raise their questions privately to the moderator, which may be suitable for highly sensitive conversations.

Although it may initially seem like a good idea to have students unmute themselves to ask a question during the panel, the conversation has the potential to get disorderly and make it difficult for both moderators and panelists to navigate the conversation. Furthermore, some students are more vocal and less shy, and may have the propensity to dominate airtime. Having the moderator field questions to raise to the panelists ensures equity in an online format. Lastly, the facilitator of the panel can copy all unanswered questions that were posted in the chat and provide students with an answer asynchronously after the conclusion of the panel.

### Tip 9: Start with a warm welcome and end with gratitude

In the online setting, deliberate reminders of humanity are important to set the tone for both patients and the students. It can be intimidating for a patient to speak in front of hundreds of students – especially in front of a blank screen. We have found that the standard practice of having students turn on their cameras at the beginning of each session and wave hello to the patient panelists establishes rapport between the patients and the student audience. It allows both the patients and the students to start the session with an acknowledgement of the other’s humanity. Similarly, at the end of the session, after connecting the panel discussion to the goals of the session, the moderator should thank the patients for their time and their stories, and then ask the students to turn on their cameras once again to wave good-bye to the panelists. This is a way for the students to express their gratitude to the patients for their teaching, and gives the patients a visual reminder of the gratitude of the students. Students can also be reminded to post comments of gratitude in the chat thread, which can later be shared with the panelists. 

### Tip 10: Have a back-up plan

Even with an abundance of careful planning, things may go awry. Technological platforms fail spectacularly, often during the least expected times. Be prepared for these eventualities. Provide panel members with call-in numbers for the session, so that they can still connect even if their Internet fails. Use the pre-established direct line of private communication to help troubleshoot these technical issues.

Planning sessions with more than one patient will lessen the impact of last-minute patient scheduling conflicts and/or patients who are dropped from the session due to a technological issue. In the event that a patient panel must end abruptly, ensure that the learning objectives for the session are shared with the students and provide back-up instructional materials and resources to allow students to fill-in the gaps for the session. 

## After the session 

### Tip 11: Debrief the session

Effectively debriefing the session immediately after its conclusion with the invited patient panelists serves several critical purposes. The incidental debriefing with other panelists, which would occur naturally after an in-person session ends, needs to be intentionally planned when conducting a virtual session. First, it enables identifying and mitigating any potential harm to the patients ( Dijk *et al*., 2020). The psychological and emotional aspects of the disease that participants have brought into the conversation with students may need to be further discussed to provide immediate support and/or resources ( Jha *et al*., 2009a). In addition, ask participants how they felt during the sessions. Were there any issues that arose, including insensitive questions or comments from students, faculty or other panelists? If so, co-producing several strategies with participants to address in the moment and prevent during future virtual panels can be accomplished.

Second, the debrief enables conversation regarding whether the learning objectives were met, from both the faculty and participant perspectives, to maximize the impact of the patient voice ( Jha *et al*., 2009b). For example, if a patient panelist dominated the session, or perhaps provided too much tangential information, the debriefing can provide them with feedback to improve the achievement of learning goals during the panel’s next iteration ( Jha *et al*., 2009b). Alternately, perhaps patients felt the faculty member dominated the session and they needed more time to share their stories fully. Actively engaging patients in these feedback conversations immediately after the panel can help resolve any interpersonal tension and support a sustainable relationship. Inquiring whether patients would consider participating again may also help in understanding whether any unresolved concerns remain. If they would not participate again, for example, clarifying the sources of their hesitancy may identify a need that remains unaddressed.

Lastly, rapid feedback on any operational issues is beneficial to further the partnership with student participants. This discussion might include training, communication, reminders, or other logistical considerations to improve the quality of the participant experience. The debrief should also be recorded, if agreeable, and/or notes can be taken to be considered for future, similar panels.

### Tip 12: Express your appreciation to the patient panelists for their participation

Understanding why patients choose to participate in medical education sessions assists faculty in longitudinally supporting sustainable patient involvement. Patient participation acknowledges their expertise and enhances their sense of empowerment and self-esteem ( Jha *et al*., 2009a; Wykurz & Kelly, 2002). Participation alongside other patients, too, provides support and companionship with peers, as well as raising their awareness of certain neglected aspects of the disease ( Dijk *et al*., 2020; Watts *et al*., 2015). Patients also have the unique opportunity to meaningfully contribute to medical education and experience personal fulfillment by doing so ( Dijk *et al*., 2020).

Being mindful to provide demonstrable ways to show participants their impact is particularly useful. These might include hand-written cards or thank you emails from students or sharing the chat thread where students expressed their gratitude to patients illustrating the impact of their participation. If the budget permits, an honorarium or a small token of appreciation would also be appropriate. At our institution, for example, we have been mailing patient panelists institutional gifts to their home (e.g., blanket, mug, or shirt with our institutional logo) to thank them for lending their time and expertise to our curriculum.

## Conclusions

Successful integration of virtual patient panels requires a clear strategy to thoughtfully execute a session that maximizes the educational experience for the students, while providing an engaging and psychologically safe experience for invited patient panelists.

Educators will benefit from an organized approach for transitioning a patient panel to an online format. The tips outlined in this paper will help guide educators in the health professions maximize their success in designing a virtual patient panel. Our approach considers factors that ensure the quality and intimacy of patient narratives that can complement course content. By carefully considering planning logistics, technology and platform features, facilitator roles, patient training, pre-briefing and debriefing, and general online etiquette, educators can maximize the educational value of a virtual patient panel.

## Data Availability

No data are associated with this article.

## References

[ref-1] Colbert CY Mirkes C Cable CT : The patient panel conference experience: what patients can teach our residents about competency issues. *Acad Med.* 2009;84(12):1833–1839. 10.1097/ACM.0b013e3181bf27db 19940596

[ref-2] Dijk SW Duijzer EJ Wienold M : Role of active patient involvement in undergraduate medical education: a systematic review. *BMJ Open.* 2020;10(7):e037217. 10.1136/bmjopen-2020-037217 32718925 PMC7389514

[ref-3] Gordon M Gupta S Thornton D : Patient/service user involvement in medical education: A best evidence medical education (BEME) systematic review: BEME Guide No. 58. *Med Teach.* 2020;42(1):4–16. 10.1080/0142159X.2019.1652731 31518544

[ref-4] Haras C Calhoun A Olson AP : Mindful medical education online. *Med Sci Educ.* 2021;31(2):863–872. 10.1007/s40670-021-01253-7 33688449 PMC7932687

[ref-5] Jackson A Blaxter L Lewando-Hundt G : Participating in medical education: views of patients and carers living in deprived communities. *Med Educ.* 2003;37(6):532–538. 10.1046/j.1365-2923.2003.01535.x 12787376

[ref-6] Jha V Quinton ND Bekker HL : Strategies and interventions for the involvement of real patients in medical education: a systematic review. *Med Educ.* 2009a;43(1):10–20. 10.1111/j.1365-2923.2008.03244.x 19140994

[ref-7] Jha V Quinton ND Bekker HL : What educators and students really think about using patients as teachers in medical education: a qualitative study. *Med Educ.* 2009b;43(5):449–456. 10.1111/j.1365-2923.2009.03355.x 19422492

[ref-8] Leeds FS Sommer EM Andrasik WJ : A Patient-Narrative Video Approach to Teaching Fibromyalgia. *J Med Educ Curric Dev.* 2020;7:2382120520947068. 10.1177/2382120520947068 32821851 PMC7412912

[ref-9] Lenton C Storr E : Patients as teachers for undergraduates: real-life experiences of long-term conditions. *Educ Prim Care.* 2015;26(2):109–112. 25898302

[ref-10] Salerno-Kennedy R Henn P O’Flynn S : Patients with chronic diseases as partners in medical education. *Clin Teach.* 2009;6(3):155–159. 10.1111/j.1743-498X.2009.00282.x

[ref-11] Stacy R Spencer J : Patients as teachers: a qualitative study of patients' views on their role in a community-based undergraduate project. *Med Educ.* 1999;33(9):688–694. 10.1046/j.1365-2923.1999.00454.x 10476021

[ref-12] Towle A Bainbridge L Godolphin W : Active patient involvement in the education of health professionals. *Med Educ.* 2010;44(1):64–74. 10.1111/j.1365-2923.2009.03530.x 20078757

[ref-13] Vail R Mahon-Salazar C Morrison A : Patients as teachers: an integrated approach to teaching medical students about the ambulatory care of HIV infected patients. *Patient Educ Couns.* 1996;27(1):95–101. 10.1016/0738-3991(95)00793-8 8788753

[ref-14] Voelker R : Virtual patients help medical students link basic science with clinical care. *JAMA.* 2003;290(13):1700–1. 10.1001/jama.290.13.1700 14519694

[ref-15] Watts L Mcpherson T Robson J : Patient experiences of participation in a medical student teaching workshop. *Med Teach.* 2015;37(1):94–96. 10.3109/0142159X.2014.947946 25154410

[ref-16] Wykurz G Kelly D : Developing the role of patients as teachers: literature review. *BMJ.* 2002;325(7368):818–821. 10.1136/bmj.325.7368.818 12376445 PMC128951

