# Supplementary material for: Practical tips for moving your patient panel online
Source: MedEdPublish (2016). 2023 Mar 24;13:19. [Version 1] doi: 10.12688/mep.19613.1 (PMC10113798; doi:10.12688/mep.19613.1)
Supplement: Peer review history

# Peer review: Reviewer response for version 1

**DOI:** 10.21956/mep.21012.r33042

**Published:** 2023-04-18

**Authors:** Anahieta Kalantari

**Affiliations:** 1 Department of Emergency Medicine, Penn State Health Milton S Hershey Medical Center, Hershey, PA, USA

General comments

 Thank you so much for providing me the opportunity to review and comment on your body of work. Any suggestions or comments I provide are done from a place to help improve your work even further. Overall, I found this as an easy read that provided a lot of practical insight on how to create an online patient panel. It is relevant to the current climate of medical education. It was quite thorough. 

 Abstract and Title

- Title suggests this is an article to help educators use an online platform for patient panels
- Abstract is short and concise. It suggests this article will offer suggestions on how to successfully move patient panels online while still providing relevant education.

Introduction

- Very concise. Nice presentation of available evidence to utilize patient panels.  Smooth transition between paragraphs. Strong reasons for maintaining an online panel.

Tip 1

- Last sentence of second paragraph is difficult to read. I had to read it twice to understand what was being conveyed. Please consider separating into 2 separate sentences or reword this one.
- Appropriate use of examples.

Tip 5

- Prepping patients for use of technology and the panel is an excellent tip. How does one do this? In tip 4, the authors mention some patients may not have the knowledge or resources for access to items allowing them to participate in patient panels. For example, a facilitator can’t zoom someone about zoom if they don’t have what they need to zoom. If this is the case, how can they be prepped.  Providing an example would assist readers and give additional tools on how to achieve tip 4.

Tip 9

- Suggestions or tips for when a student is unwilling to cooperate?

Conclusion

- Concise and appropriate

If evidence from practice is presented, are all the underlying source data available to ensure full reproducibility?

Yes

Is the topic of the practical tips discussed accurately in the context of the current literature

Yes

Are all factual statements correct and adequately supported by citations?

Yes

Are the conclusions drawn balanced and justified on the basis of the presented arguments?

Yes

Are arguments sufficiently supported by evidence from the published literature and/or the authors’ practice?

Yes

Reviewer Expertise:

medical education, physician wellness, equity and inclusion

I confirm that I have read this submission and believe that I have an appropriate level of expertise to confirm that it is of an acceptable scientific standard.

# Peer review: Reviewer response for version 1

**DOI:** 10.21956/mep.21012.r33041

**Published:** 2023-03-31

**Authors:** Arnyce R. Pock

**Affiliations:** 1 Department of Medicine, Uniformed Services University of the Health Sciences, Bethesda, MD, USA

Although it appears that this paper may have originally been prepared for submission to *Medical Teacher* as part of their "12 Tips" compendium, the paper highlights twelve practical suggestions for designing and facilitating an interactive, online presentation by a patient panel.

 The article is nicely written, but in terms of constructive critiques, three items come to mind.  First, the title "moving your patient panel online" could be perceived as being somewhat misleading, as the term "patient panel" is often equated with the list of patients empaneled to a clinician in his/her/their clinic. Perhaps simply re-phrasing the title and/or abstract to clarify that the focus is on describing how an online format could be used for engaging patient panelists or for conducting an interactive panel of patient-educators, would make the focus a bit clearer.

 As for the tips themselves, they are clearly written, practical, and actionable. That said, Tip 1 might benefit from including a reference to pre-briefing the patient panelists, so that they too, are aware of the goals of the session. Moreover, it can often be helpful to prep the patient panelists by sharing a list of likely questions with them in advance, so they can have some time to reflect and consider their replies, in advance.

 The third is just a technical note, but Tip 9 suggests that students should "...turn on their cameras...to wave goodbye to participants," at the end of the session. This is certainly appropriate, but it seems to contradict Tip 8, which indicates that students should be wearing professional attire and have their cameras on, to demonstrate their attention and engagement. i.e. If students have their cameras on throughout the presentation--demonstrating their engagement, it really shouldn't be necessary for them to turn their cameras back on at the end of the session to wave farewell.

If evidence from practice is presented, are all the underlying source data available to ensure full reproducibility?

Yes

Is the topic of the practical tips discussed accurately in the context of the current literature

Yes

Are all factual statements correct and adequately supported by citations?

Yes

Are the conclusions drawn balanced and justified on the basis of the presented arguments?

Yes

Are arguments sufficiently supported by evidence from the published literature and/or the authors’ practice?

Yes

Reviewer Expertise:

Undergraduate Medical Education and Curricular Reform

I confirm that I have read this submission and believe that I have an appropriate level of expertise to confirm that it is of an acceptable scientific standard.
